# Non-Smoking-Associated Lung Cancer: A Distinct Entity in Terms of Tumor Biology, Patient Characteristics and Impact of Hereditary Cancer Predisposition

**DOI:** 10.3390/cancers11020204

**Published:** 2019-02-10

**Authors:** Elisabeth Smolle, Martin Pichler

**Affiliations:** 1 Division of Pulmonology, Department of Internal Medicine, Medical University Graz, 8036 Graz, Austria; elisabeth.smolle@medunigraz.at; 2 Department of Experimental Therapeutics, Division of Medicine, The UT MD Anderson Cancer Center, Houston, TX 77030, USA

**Keywords:** non-small cell lung cancer, non-smoker, tumor microenvironment, targeted treatment

## Abstract

Non-small cell lung cancer (NSCLC) in non-, and especially in never-smoking patients is considered a biologically unique type of lung cancer, since risk factors and tumorigenic conditions, other than tobacco smoke, come into play. In this review article, we comprehensively searched and summarized the current literature with the aim to outline what exactly triggers lung cancer in non-smokers. Changes in the tumor microenvironment, distinct driver genes and genetic pathway alterations that are specific for non-smoking patients, as well as lifestyle-related risk factors apart from tobacco smoke are critically discussed. The data we have reviewed highlights once again the importance of personalized cancer therapy, i.e., careful molecular and genetic assessment of the tumor to provide tailored treatment options with optimum chances of good response—especially for the subgroups of never-smokers.

## 1. Introduction

Lung cancer is the second most common incident cancer diagnosis in men, and the fourth most common cancer diagnosis in women, accounting for most cancer-related deaths in both men and women, with 1.7 million global deaths a year [1,2,3]. More than 85% of lung cancer cases are related to a positive history of smoking (i.e., smoking-related or smoking-associated lung cancer). Smoking leads to an accumulation of genetic alterations in oncogenes and tumor suppressor genes ultimately causing cancer [4]. Previous reports and literature reviews have addressed the topic of lung cancer in non- and never-smokers [5,6,7,8,9,10,11,12], and here we aim at providing a more updated review by going into detail also with molecular, immunological and genetic aspects. In Asian countries the proportion of never-smoking lung cancer patients is generally higher (up to 15%) [8]. Epidemiologic studies show that never-smoking lung cancer patients are more often female, show an adenocarcinoma (AC) histologic subtype and are often of East Asian ethnicity. Within the last decade, genome-wide studies clearly indicated that the underlying tumor biology in lung cancers of non-smokers (meaning never-smokers and patients with a negligible history of smoking and small likelihood that the tumor was smoking-related) differs dramatically from smoking-related lung cancer, featuring a different pattern of molecular alterations [10,13,14,15,16,17]. Another interesting finding in non-smoker lung cancer is the fact that patients are significantly younger, have a better prognosis and respond to treatment better than smokers with lung cancer [5,7,9,18]. The main reason for the favorable outcome in non-smokers is the occurrence of certain molecular subtypes (oncogene-addicted lung cancer), enabling the specific treatment with Previous epidermal growth factor receptor-tyrosine kinase inhibitors (EGFR-TKIs) or other agents [19,20]. Such genetic alterations and patterns of mutation that are specific for lung cancer have primarily been outlined for AC, whereas no genetic mutations have been linked to squamous cell carcinoma (SCC) specifically, especially not for non-smokers [4].

Generally spoken, environmental tobacco smoke at home or at the workplace [21], radon [22], cooking oil vapor [23], indoor coal burning, hormonal replacement therapy [20], exposure to asbestos/heavy metals [6], infectious factors and air pollution have been linked to lung carcinogenesis in non-smokers [24,25].

It has also been proposed that lung cancer in smokers versus non-smokers is characterized by a distinct tumor microenvironment [26]. Tobacco smoke causes DNA damage in cells of the bronchial epithelium which causes dysfunction in the immune system of the lung. Immune cells that infiltrate the tumor are likely to influence survival, and possibly response to treatment. However, the role of the tumor microenvironment with a special emphasis on immune cells has not been widely studied yet [26]. In the next sections, we will highlight and discuss the knowledge of distinct molecular and epidemiological differences between lung cancer in never-smokers with a special emphasis and separation between different lung cancer histology.

## 2. Squamous Cell Carcinoma in Non- or Never-Smokers

In 2017, Park and colleagues performed a study to identify potential genetic alterations specific for squamous cell carcinoma (SCC) in non-smokers [4]. For that purpose, an Array comparative genomic hybridization (ArrayCGH) analysis was conducted in 19 patients suffering from SCC. Previous CGH analyses have shown that amplification of chromosome 3q25-qter frequently occurs in SCC [27]. Among the 19 SCC patients that were studied by Park et al., there were eight non-smokers compared to 11 smokers. Sixteen gene regions were significantly altered, according to ArrayCGH. Three gain (5p15.33, 8q24.21, and 11q13.3) and four loss regions (4q35.2, 9p21.3, 10q23.31, and 15q11.2) were found, that also overlapped with data from The Cancer Genome Atlas (TCGA) which contains data on copy number variations in SCC [28]. The investigators identified 15 genes within the significantly altered regions, that have also been reported in the Cancer Gene Census (*ATM, CCND1, CDKN2A, DUX4L1, EZH2, FOXP1, LRIG3, MEN1, MITF, NRG1, NUMA1, PTEN, TERT, WHSC1L1, and WRN*) [29]. The proto-oncogene *GAB2* (11q14.1) was found to be frequently amplified in non-smoking patients [4]. To secure this finding, protein expression of GAB2 was investigated by means of immunohistochemistry, and the protein was also found to be upregulated in tissues of non-smokers as compared to smokers (37.5% vs. 9.0%, *p* = 0.007) [4]. Thus, *GAB2* amplification is likely to contribute to SCC development in the subgroup of non-smokers, and may also serve as a biomarker in the near future.

An interesting case report of a non-smoker female patient with SCC of the lung favors a genomics-, proteomics- and metabolomics-based approach to treatment, highlighting the importance of personalized medicine especially in non-smoking individuals [30]. The Caucasian female patient developed SCC in the absence of smoking, and with no history of asbestos exposure. The patient’s father, who was a smoker, died from lung cancer at age 63; apart from that, no family history of cancer was known. The tumor was surgically removed shortly after diagnosis, and at pathologic examination the SCC featured a unique, mainly perialveolar and perivascular growth pattern. There was positive immunostaining for p63 and cytokeratin (CK) 5/6, whereas CK7, thyroid transcription factor 1, synaptophysin and chromogranin were all negative. Ki67 proliferation marker immunostaining showed 20 percent positive cells [30]. Interestingly, this patient had undergone surgical resection of a SCC of the head and neck region two years prior to diagnosis of the lung SCC. According to in-depth pathological assessment it was stated that these two tumors were two distinct entities of SCC, and that the lung tumor was not recurrent disease of the head and neck SCC. The patient is still alive two years after the lung tumor resection, and four years after the resection of the SCC at the neck. In this special case, analysis of certain somatic driver mutations was carried out. A predominance of C>T transitions in tumoral lung tissue was found, not corresponding to the specific cancer signature usually correlating with tobacco smoking (characterized by an abundance of C>A transversions). Pathway analysis showed that mutations in the SCC tissue predominantly affected genes involved in extracellular matrix organization (*p* = 0.005), transmembrane transport of small molecules (*p* = 0.010) and collagen formation (*p* = 0.034). Mutations in these pathways have previously been reported in a study on whole exome sequencing in lung cancer [31]. Interestingly, in this case no mutations in the most frequent lung cancer driver genes, namely *EGFR, KRAS, AKT* and *ROS1* were present in the tumor sample, and neither was the *EML4-ALK* fusion gene [30]. The authors then used a combination of next generations sequencing (NGS) techniques to test the hypothesis that in this case of a never-smoking female, the lung carcinoma was of oligogenic origin. Among the 11 germline-mutated cancer-related genes, two (*ACACA* and *DEPTOR*) are known to be associated with common driver genes: *ACACA* is associated with *BRCA1*, and *DEPTOR* with *EGFR*. One particular missense variant in the *ACACA* gene (c.C1948T, p.Arg650Trp (NM_198837.1, exon 16)) has been predicted as deleterious, leading to a loss of function of Acetyl-CoA carboxylase alpha which is a crucial enzyme for long-chain fatty acid synthesis [32]. The authors of this case report conclude that both primary SCC tumors which the patient developed were triggered by a special oligogenic germline signature consisting of at least 11 mutations, two of them leading to the activation of mTOR and *BRCA1* [30]. A proteomic/genomic/metabolomic sequencing approach is thus particularly useful to find personalized treatment strategies and accurate estimations of prognosis, especially in patients that lack common risk factors for a certain cancer species, e.g. tobacco smoking for lung cancer. However, it must be pointed out that this report has a clear limitation, because tissue from only one individual was analyzed. In the future, more sequencing data of tissue samples from never-smoker lung cancer patients would be of use to find out more about genetic patterns in this special subgroup of patients.

In another interesting study, the effect of the programmed death 1 (PD-1)-receptor targeting checkpoint inhibitor nivolumab in never-smokers with advanced squamous non-small cell lung cancer was investigated [33]. Data on the general response to immunotherapy in non-smokers is controversial: some studies have shown better response rates, whilst other analyses showed that never-smokers seem to benefit less from immunotherapy than smokers. In this study, the authors aimed to analyze a cohort of never-smokers with advanced SCC in-depth with respect to their response to nivolumab. Nivolumab was administered in 371 patients at a dosage of 3 mg/kg every 2 weeks for a maximum of 24 months, and safety was monitored [33]. Among the cohort there were 31 never-smokers (8%). The objective response rate, disease-control rate, and the median overall survival were 23%, 45%, and 12.1 months (95% confidence interval: 3.7–20.4), respectively, in never-smokers, and 18%, 47%, and 7.9 months (95% confidence interval: 6.2–9.6), respectively, in the whole population analyzed. Any-grade and grade 3–4 treatment-related adverse events (AE) were reported in 12 (39%) and 3 (10%) never-smokers, respectively, and in 109 (29%) and 21 (6%) patients of the total group, respectively. Treatment had to be discontinued due to side effects in 4 non-smokers, and in 26 patients overall [33]. Summing up this report, in the pre-treated never-smokers suffering from advanced SCC, safety and efficacy of nivolumab treatment were similar and consistent to the overall study population as well as to previous reports on nivolumab. According to this analysis, there is no evidence that never-smokers might benefit less from nivolumab as compared to smokers.

## 3. Adenocarcinoma in Non- or Never-Smokers: Patient Characteristics

Starting again with a case report, we discuss an article about a never-smoker female lung cancer patient with multifocal lung AC, where morphological and genetic heterogeneity was assessed [34]. The patient presented with three lung nodules occurring at different time points, which were surgically removed. Unfortunately, the patient relapsed, and was subsequently treated with an EGFR-directed tyrosine kinase inhibitor (TKI), since *EGFR* exon 21 mutation had been detected. Progression free survival upon treatment with the TKI lasted for three months and was continued for six months, until clinical progression [34]. Tumor samples were then analyzed by means of a 30-gene NGS-gene panel, allowing for the evaluation of intra- and inter-tumor heterogeneity. Interestingly, the three lung tumors were confirmed independently according to NGS. The synchronous tumor samples featured different molecular profiles. Identical *EGFR, PIK3CA* and *TP53* mutations were found in one of the three primary tumors and in the metastasis that occurred later [34]. The patient in this case report may have a certain genetic cancer predisposition, which may explain the independent lung ACs, limited response to treatment and the fact that she was a never-smoker. In a review article by Okazaki et al., it was stated that genes commonly associated with the metabolic syndrome overlapped with genes frequently mutated in AC in never smokers [35]. Moreover, the incidence of AC is obviously increasing worldwide, and generally AC is more prevalent in never-smokers than any other histologic lung cancer type. All these findings lead to further questions, e.g. whether lung AC is a different disease than non-AC lung cancers, why there is an obvious female predisposition and what prevention strategies exist [35]. 

Recently, Li and colleagues conducted an integrative analysis where they included 11 lung cancer gene-expression datasets that provide data from 1111 lung AC and 200 samples of adjacent normal tissue [26]. According to this study, distinct pathways were altered in ever-smokers, and different pathways in never-smokers. Never-smokers had a better outcome as well. In the course of this study, compositional patterns of 21 types of immune cells in lung AC were characterized, revealing complex and multilayered associations between the composition of immune cell subtypes and clinical outcome [26]. Two subsets of immune cells, namely mast cells and CD4+ memory T cells were found to have completely opposite associations with outcome in resting, as compared to activated status. Resting mast cells (defined by not having undergone degranulation), which were found to be decreased in numbers in tumor samples, compared with adjacent normal tissue, were predictors of a favorable outcome, but macrophages, activated mast cells (mast cells after degranulation) and activated CD4+ memory T cells that were enriched in the carcinoma samples predicted a poor prognosis [26]. Differences in the composition of immune cell types were found in never- and ever-smokers: there were more resting mast cells in never-smokers, and more resting CD4+ memory T cells as well, these being associated with a better outcome. In ever-smokers, there were more activated mast cells and CD4+ cells, which correlated with a generally worse prognosis. What’s more, a variety of chemokines and associated chemokine receptors (e.g., the CKCL11-CXCR1 axis) were selectively mutated in smoking-associated lung cancers, and these alterations also correlated with the status switch of immune cells from resting to the activated form. Taken together, these findings indicate unique changes in the lung cancer microenvironment that are caused by tobacco smoke, altering the intrinsic immune system of the bronchi. It is thus very likely that certain patterns of immune cell dysfunction lead to a worse prognosis especially in lung cancer patients who continue smoking [26].

The role of metabolic syndrome in lung AC, especially in non-smoking patients, is currently under intensive exploration: Yang and colleagues did a survey on body mass index (BMI) and waist circumference in a prospectively studied population of women aged 55–69 years, who were followed up for 13 years. It was significant (*p* < 0.15) that patients with lung cancer had higher waist circumferences [36]. According to another investigation where the pattern of adiponectin quantitative trait loci (QTLs) in association with gene expression correlation was analyzed, genes related to metabolic syndrome were found also to contribute to cancer formation [37]. *EGFR*, *VTL1A*, *TNFRSF10C*, *C3ORF21* and hyper-methylation of *TNFSF10C*, *BHLHB5*, and *BOLL* are involved in both lung AC formation and pathways related to metabolic syndrome, according to genome wide association studies (GWAS) [5,35]. The link between the metabolic syndrome and lung AC in non-smokers is also supported by Mazieres and colleagues who examined 140 female AC patients, amongst them 63 never-smokers and 77 former or current smokers [38]. In never-smokers, histology showed lipidic features (lipid droplets inside cancer cells; not to be confounded with a lepidic growth pattern of NSCLC) significantly more often (60.3% vs. 37.7%, *p* = 0.008) as compared to smokers. It is common knowledge that obesity, a predominantly sedentary lifestyle, too much alcohol consumption and a diet high in—especially saturated fatty acids - lead to metabolic syndrome but is also associated with a higher incidence of malignant disease in general [39,40]. A frequent complication of metabolic syndrome is type-2 diabetes, which increases the risk of lung cancer, most of all for females (relative risk for women with diabetes = 1.14) [41].

## 4. Physical Inactivity

Cannioto et al. investigated the association of physical inactivity with lung cancer [42]. Since it has been proven for many types of malignant disease that lifetime inactivity goes along with an increased cancer risk, the authors wanted to show whether this holds true also for lung cancer, independently of other risk factors such as smoking. In this hospital-based, case-control study, data from 660 lung cancer patients and 1335 matched controls, who did not suffer from any malignant disease, were analyzed [42]. Multivariate logistic regression analysis was used to assess the association between a primarily sedentary lifestyle and the risk of lung cancer. Furthermore, Cox proportional hazard models were utilized for a calculation on how closely lifetime physical inactivity and mortality among lung cancer patients is related. Not surprisingly, a significant positive correlation of physical inactivity and lung cancer risk was observed [Odds ratio (OR) = 2.23, 95% confidence interval (CI): 1.77–2.81]. Among never-smokers (OR = 3.00, 95% CI: 1.33–6.78) and former smokers (OR = 3.00, 95% CI: 1.33–6.78) the association was significant as well. The authors also described a significant positive correlation between lifetime physical inactivity and the mortality from lung cancer [Hazard ratio (HR) = 1.40, 95% CI: 1.14–1.71]; here the association remained significant also for the non-smoking patients [42]. A different study published in 2017 also evaluated the impact of physical activity on lung cancer risk. It has already been shown in numerous studies that regular physical activity decreases lung cancer risk; the risk reduction has been reported to range from 20 to 50% when the most active study participants were compared to the least active individuals [43]. Being either underweight or obese also increases lung cancer risk, following a nonlinear inverted U-shaped relation [43]. It has to be kept in mind though, that an active lifestyle and regular exercise often means less likelihood to engage in smoking. Thus, Patel and colleagues especially stratified for smoking status when investigating physical activity and lung cancer; also because smokers tend to be not only less active, but on average also have a lower body-mass-index as compared to non-smokers. Data of 162679 men and women from the American Cancer Society Cancer Prevention Study-II Nutrition Cohort were analyzed, who were all free of cancer at enrollment in this study (1992–1993) [44]. Baseline physical activity (MET-hours per week; none, 0.1 to < 8.75; 8.75–17.4; >17.5 MET-hours/week), baseline body mass index and waist circumference were assessed in relation to lung cancer risk [43]. Risk stratification for smoking history, years since quitting among former smokers and adjustment for other possible confounders was carried out. During the follow-up time of 2,384,546-person years, 4669 men and women were diagnosed with lung cancer (453 never smokers, 1452 current smokers and 2764 ex-smokers) [43]. Interestingly, physical activity was not associated with lung cancer risk in this large analysis within any of the smoking strata, except in former smokers who quit less than 10 years ago (RR = 0.77; 95% CI 0.67–0.90 for >17.5 MET hours/week). BMI was inversely associated with lung cancer risk in a similar way, also in the former-smoking group who quit <10 years (RR = 0.68; 95% CI 0.55–0.84 for >30 kg/m2). The authors clearly conclude that—although evidently protective against a variety of cancer subtypes—physical activity may not lower the risk for lung cancer [43].

## 5. Asbestos and Radon

Although uncommon causes of lung cancer per se, occupational carcinogens and radon can sometimes contribute to lung carcinogenesis. In the literature, no data on this topic addressing specifically the non-smoking population, can be found—most probably due to the fact that most cases of lung cancer where occupational carcinogens also play a role, do occur in individuals who smoked as well. A study has been done on the occurrence of radioactive radon gas, generated from uranium and thorium in underlying rocks and seeps, in Norwegian buildings [45]. Radon gas and its decay products emit radiation that promotes lung carcinogenesis, and in people exposed to radon, this is considered the second most important risk factor for lung cancer, directly after tobacco smoke. In Norway, average radon concentrations in buildings are higher than in most industrialized countries. Hassfjell et al. have estimated the radon-related risk of lung cancer in Norway, using data from the largest pooled European analysis of case-control studies, combined with the largest set of data on radon concentration measurements in Norwegian homes. According to this data, it was calculated that radon gas contributes to about 12% of all cases of lung cancer annually in Norway; meaning that in the year 2015 for instance, in 373 cases of lung cancer radon was a contributory factor [45]. However, the authors clearly state that in most lung cancer cases, former or current tobacco smoking was still the main risk factor.

Asbestos is an occupational carcinogen that also endorses lung cancer formation, though asbestos is usually known for causing pleural mesothelioma. Accumulating evidence has highlighted the role of epigenetic deregulation caused by asbestos exposure, and thus in 2017 Kettunen et al. did a genome-wide DNA-methylation analysis, investigating the impact of asbestos on DNA methylation [46]. The researchers used "Illumina HumanMethylation450K BeadChip" for methylation analysis in 28 samples of lung cancer tissue. Also, in this study, the majority of patients investigated were smokers [46]. Differentially methylated regions (DMR), as well as differentially methylated CpGs (DVMC) were identified, with individual CpGs being evaluated in-depth by pyrosequencing in an independent series of 91 NSCLC samples and corresponding normal lung tissue. *BEND4, ZSCAN31* and *GPR135* were found to be significantly hypermethylated in asbestos-associated NSCLS. DMRs in the genes *RARB*, *GPR135*, and DVMCs in the genes *NPTN*, *NRG2* and *GLTs5D2* (amongst others) were significantly associated with asbestos exposure (comparing exposed vs. not-exposed tumors). The authors of this study also compared DVMCs related to asbestos or positive smoking history, and found that 96% of the elements were unique to either of the exposures, suggesting that the methylation pattern is strongly influenced by the specific risk factor. This data suggests, that epigenetic changes may be influenced by environmental risk factors very strongly, and that asbestos causes different changes than tobacco smoke alone [46]. Another interesting analysis aimed at outlining altered micro RNA (miRNA) expression in NSCLC upon exposure to asbestos [47]. Generally, it is known that altered miRNA expression is an early step in carcinogenesis when occupational and environmental carcinogens come into play. The authors sought to identify an asbestos-related profile of miRNA changes, able to discern asbestos-induced NSCLC from cancer with a different etiology. Four groups of patients were included in this study: those with asbestos-related NSCLC, asbestos-unrelated NSCLC, subjects with malignant pleural mesothelioma, and healthy individuals. Four serum miRNAs (miR-126, miR-205, miR-222 and miR-520g) were significantly associated with asbestos-related NSCLC, or mesothelioma [47]. Increased expression of miR-126 and miR-222 are both involved in major cancer-promoting pathways. The authors suggest that epigenetic changes caused by asbestos, as well as cross-talk between cancer- and stroma-cells could lead to the repression of miR-126 which promotes tumor growth, angiogenesis and invasion. It is concluded that miRNAs are potentially involved in asbestos-related malignant disease, influencing specific mechanisms whereby asbestos promotes cancer formation—and these mechanisms may also differ from the conventional tobacco-smoke related ones.

## 6. Immunological Changes: Tumor Microenvironment in Never-Smokers

In never-smokers, the immunologic homeostasis within the tumor microenvironment seems to be less compromised when compared to ever-smokers [26]. Notably, not all immune cells impact lung carcinogenesis in a similar way: inflammatory cells are recruited into the lung as a result of tobacco smoking. On the one hand these cells are helpful because they are trying to minimize the damage which is done by the carcinogenic substances, on the other hand, however, the immune cells may weaken the bronchial epithelial cells and cause harmful pro-inflammatory and immune reactions [48]. When a tumor arises, the immune cells are also part of the harmful tumor microenvironment and can even contribute to tumor growth, invasion and metastatic spread [49]. This is proven by certain biologicals that have recently been established as targeted treatment options for lung cancer, targeting for instance cytotoxic T-lymphocyte-associated antigen 4 (CTLA-4) (ipilimumab), or PD-1-receptor and PD-1 ligand (PD-L1) which are targeted by nivolumab and atezolizumab [49]. It has been reported according to a study published in 2018, that 17 out of 20 investigated pathways of carcinogenesis, related to immune response, were altered in a different way in AC of never-smokers as compared to ever-smokers [26]. Compositional differences of 14 kinds of immune subsets between tumors and normal samples were reported, and the composition of leukocyte subtypes correlated strongly not only with smoking history, but also with outcome [26]. M0 macrophages and total macrophage count strongly correlated with a poor prognosis. Furthermore, the immune score of CD8+ T cells was associated with a more favorable prognosis. Although it has been shown in ovarian- and breast cancer that CD8+ T cells usually mean a better outcome [50], in lung AC the results were controversial: in stage IV NSCLC patients undergoing chemotherapy, CD8+ T cells also correlated with a better prognosis [51], but some other studies suggested no influence of CD8+ T cells on NSCLC survival whatsoever [52,53]. Numerous studies have already shown how strongly immune reactions are associated with carcinogenesis. Either the immune system is capable to protect against cancer progression, or it can enhance tumor growth, invasion and metastasis by negatively influencing the tumor microenvironment and weakening the surrounding healthy cells. Thus, it is very likely that smoking, which obviously alters the immune system in the bronchi long before carcinogenic effects are observed, allows for specific changes in the cancers of smokers, featuring ultimately a different microenvironment as compared to lung cancers of never-smokers.

## 7. Anaplastic Lymphoma Tyrosine Kinase -Rearrangement in Lung Adenocarcinoma in Non- and Never-Smokers

Targeted therapy has become a well-established therapeutic tool for the treatment of lung cancer. In tumors featuring anaplastic lymphoma tyrosine kinase (*ALK*)-rearrangement, agents like crizotinib, ceritinib, alectinib, brigatinib and lorlatinib are valid treatment options, hence a correct molecular profiling of newly diagnosed tumors is of great importance [54,55]. Frequently, *ALK* rearrangements result from inversions on chromosome 2p [inv(2)(p21;p23)] which leads to a fusion of *ALK* with the echinoderm microtubule-associated protein-like 4 (*EML4*) gene [56]. The gold standard to evaluate ALK-rearrangement is by fluorescence in situ hybridization (FISH). In a study by Williams and colleagues, the aim was to assess the prevalence of *ALK*-rearrangements in lung AC samples of lifetime non-smokers, as well as long-term ex-smokers (quit > 10 years prior to diagnosis) [57]. According to the literature, *ALK* gene rearrangement is found in 2–5% of all non-small cell lung cancers, being more common in lifetime non-smokers with adenocarcinoma as compared to smokers with adenocarcinoma, or squamous cell carcinoma. However, accurate assessment of *ALK*-rearrangement in long-term ex-smokers has not been done before the year 2016 [57]. The authors enrolled 251 cases of resected lung AC samples, including 79 non-smokers and 172 ex-smokers who had quit smoking for over 10 years [57]. *ALK*-rearrangement was evaluated via FISH, and immunohistochemistry (IHC) as well. Four out of 251 cases featured *ALK*-rearrangement. All of these four were non-smokers. In samples of long-term ex-smokers, no *ALK*-rearrangements were observed [57]. The analysis revealed strong evidence of an increased prevalence of *ALK* gene rearrangement in the non-smoking population, as compared to the general population of lung adenocarcinoma patients. Interestingly, there was no significant difference in *ALK*-rearrangement between the ex-smokers and the general population of patients with resected lung AC. This study confirmed that ALK-rearrangement is more common in non-smoking patients suffering from lung adenocarcinoma. However, the incidence reported in this analysis is uncommonly low (5.1% in non-smokers; 1.6% overall) when compared to previous reports. This is presumably due to the circumstance that most samples were early-stage, resected lung cancers; since it has been shown that *ALK*-rearrangements tend to occur more often in advanced, mainly stage IV, cancers [58].

Overall, the occurrence of the *ALK*-translocation in lung AC of never-smokers clearly indicated that the patho-mechanism is based on a single genetic driver rather than on an accumulation of genetic lesions in a variety of cancer genes, as it is the case in smoking-related AC.

## 8. Conclusions

The data which we have summarized above indicates that NSCLC in non-, especially in never-smokers, is a distinct tumor entity featuring a different tumor biology and microenvironment as compared to tobacco associated lung carcinomas. We have summarized key findings of the studies mentioned in the table below (Table 1). 

From the epidemiological perspective, other risk factors such as metabolic disorders may play a role, as well as germline mutations that lead to cancer formation in certain individuals independent from lifestyle and exposure to carcinogens. The take-home message of this short literature review is the paramount importance of personalized medicine, in-depth molecular assessment and targeted treatment options especially in never-smoking patients suffering from lung cancer, since their tumors differ distinctly in molecular pathology, prognosis and response to treatment in comparison to "conventional" smoking-associated tumors.

## Figures and Tables

**Table 1:** Non-smoking associated lung cancer—summary of recent findings.

Tumor Type	Patient Characteristics	Aim Of The Study	Methods	Key Findings	Discussion/Conclusion	Reference
Adenocarcinoma (AC)	1111 lung AC and 200 samples of adjacent normal tissue; comparison of smokers vs. non-smokers	Characterization of tumor microenvironment/pattern of tumor-associated immune cells	Online lung cancer data analysis via Gene Expression Omnibus (GEO); to determine the fraction of immune cells in tumors, a linear support vector regression-based method, CIBERSORT, was applied to estimate the relative ratios of 21 leukocyte subtypes	Distinct pathways were altered in lung carcinogenesis in ever-smokers and never-smokers. Never-smoker patients had a better outcome than ever-smoker patients. Mast cells and CD4^+^ memory T cells were associated with poor outcome when activated compared to the resting form; cigarette smoke induced activation of these immune cells	Tobacco smoke alters the composition of immune cells in lung adenocarcinoma; activation of CD4+ memory T cells and mast cells by smoking may be responsible for the worse outcome in smokers as compared to non-smokers	[26]
AC	1 never-smoker female patient with multifocal lung AC; after surgery the patient underwent treatment with EGFR-TKI	Assessment of morphological and genetic tumorheterogeneity	30-gene next generation sequencing (NGS) panel, allowing for evaluation of intra- and inter-tumoral heterogeneity	The 3 lung tumors were confirmed independent according to NGS; identical *EGFR, PIK3CA* and *TP53* mutations were found in one of the three primary tumors and in the metastasis that occurred later on	In this non-smoker female patient, some type of genetic cancer predispositon is likely, explaining the three genetically independent lung ACs and limited response to treatment	[34]
Lung cancer (any histological type)	Prospectively studied population of women aged 55-69 years, who were followed up for 13 years	Evaluation of the role of metabolic syndrome in lung cancer	Prospective cohort study; focus on body mass index (BMI) and waist circumference	Patients with lung cancer had a significantly higher waist circumference	Abdominal obesity may increase the risk for lung cancer when stratifying for other common risk factors	[36]
AC	140 female AC patients, amongst them 63 never-smokers and 77 former or current smokers	Investigating the link of metabolic disorders and lung AC in never-smokers	Histologic analysis of tumor samples, smoking-associated vs. not smoking-associated tumors	In never-smokers, lipidic histologic differentiation was found significantly more often as compared to smokers	Non-smokers with a sedentary lifestyle, hyperlipidemia and other signs of metabolic disease might be at higher risk for lung cancer as compared to non-smokers without metabolic syndrome	[38]
Lung cancer (any histological type)	660 lung cancer patients and 1335 matched controls who did not suffer from any malignant disease	To determine whether physical inactivity increases lung cancer risk, and whether it increases mortality in case of lung cancer	Case-control study; assessment of the association of inactive lifestyle and risk of lung cancer via multivariate logistic regression analysis; Cox proportional hazard models were used for estimation of the connex of inactivity and mortality from lung cancer	Significant positive correlation of physical inactivity and risk of lung cancer; significant positive correlation between lifetime physical inactivity and lung cancer-related mortality; also significant for non-smoking lung cancer patients	Physical inactivity increases not only the risk of lung cancer but also lung cancer-related mortality	[42]
Lung cancer (any histological type)	Data of 162679 men and women from the American Cancer Society Cancer Prevention Study-II Nutrition Cohort were analyzed, who were all free of cancer at enrollment in this study (1992-1993)	Assessment of the correlation of baseline physical activity and lung cancer incidence over the follow-up period	Baseline physical activity (MET-hours per week; none, 0.1 to <8.75; 8.75-17.4; >17.5 MET-hours/week), BMI and waist circumference were assessed in relation to lung cancer risk	Physical activity was not associated with lung cancer risk, except in former smokers who quit less than 10 years ago (for >17.5 MET hours/week); BMI was inversely associated with lung cancer risk	According to this study, physical activity is not a protective factor regarding the incidence of lung cancer	[43]
AC	Never-smokers and long-term ex-smokers who quit >10 years prior to diagnosis with AC; 251 cases of resected lung AC (79 never-, and 172 ex-smokers)	To assess the prevalence of *ALK*-rearrangements in lung AC samples of lifetime non-smokers, as well as long-term ex-smokers (quit >10 years prior to diagnosis)	*ALK*-rearrangement was evaluated via fluorescence in situ hybridization FISH, and immunohistochemistry (IHC)	strong evidence of increased *ALK* gene rearrangement in the non-smoking population; no significant difference in *ALK*-rearrangement between the ex-smokers and the general population	*ALK*-rearrangement is more common in non-smoking patients	[57]
Squamous cell carcinoma (SCC)	19 patients suffering from SCC (8 non-smokers, 11 smokers)	Evaluation of genetic differences in SCC of smokers, as compared to non-smokers	Array comparative genomic hybridization (ArrayCGH); immunohistochemistry	16 gene regions were significantly altered, according to ArrayCGH in non-smokers compared to smokers; the proto-oncogene *GAB2* (11q14.1) was significantly amplified in non-smoking patients, and the GAB2 protein was upregulated as well	*GAB2* amplification is likely to contribute to SCC development in non-smokers	[4]
SCC	1 non-smoker female patient with SCC and history of a SCC at the neck 2 years prior	To determine germline cancer predisposition in this special case	Assessment of somatic driver mutations via whole exome sequencing	Predominance of C>T transitions in tumoral lung tissue; usually not found in tobacco-smoke-associated lung cancer; no mutations of frequent driver genes of lung cancer were found	In this case a special oligogenic germline signature predisposed for SCC formation; personalized medicine is especially important in non-smoker patients	[30]
SCC	371 patients with SCC; among them were 31 never-smokers	Analyzing the response to nivolumab in SCC patients who are non-smokers	Nivolumab was administered at a dosage of 3 mg/kg every 2 weeks for a maximum of 24 months, and safety was monitored	The objective response rate, disease-control rate and median overall survival were comparable in the smoking vs. the never-smoking group	Safety and effecacy of nivolumab seems to be similar in never-smokers as compared to smokers with SCC	[33]

## References

[1] Global Burden of Disease Cancer Collaboration (2017). Global, Regional, and National Cancer Incidence, Mortality, Years of Life Lost, Years Lived with Disability, and Disability-Adjusted Life-years for 32 Cancer Groups, 1990 to 2015: A Systematic Analysis for the Global Burden of Disease Study. JAMA Oncol..

[2] Siegel R., Naishadham D., Jemal A. (2013). Cancer statistics, 2013. CA Cancer J. Clin..

[3] Bray F., Ferlay J., Soerjomataram I., Siegel R.L., Torre L.A., Jemal A. (2018). Global cancer statistics 2018: GLOBOCAN estimates of incidence and mortality worldwide for 36 cancers in 185 countries. CA Cancer J. Clin..

[4] Park Y.R., Bae S.H., Ji W., Seo E.J., Lee J.C., Kim H.R., Jang S.J., Chang-Min C. (2017). GAB2 Amplification in Squamous Cell Lung Cancer of Non-Smokers. J. Korean Med. Sci..

[5] Okazaki I., Ishikawa S., Ando W., Sohara Y. (2016). Lung Adenocarcinoma in Never Smokers: Problems of Primary Prevention from Aspects of Susceptible Genes and Carcinogens. Anticancer Res..

[6] Couraud S., Zalcman G., Milleron B., Morin F., Souquet P.J. (2012). Lung cancer in never smokers–a review. Eur. J. Cancer.

[7] Sun S., Schiller J.H., Gazdar A.F. (2007). Lung cancer in never smokers—a different disease. Nat. Rev. Cancer.

[8] Thun M.J., Hannan L.M., Adams-Campbell L.L., Boffetta P., Buring J.E., Feskanich D., Flanders D.W., Jee S.H., Katanoda K., Kolonel L.N. (2008). Lung cancer occurrence in never-smokers: An analysis of 13 cohorts and 22 cancer registry studies. PLoS Med..

[9] Kawaguchi T., Takada M., Kubo A., Matsumura A., Fukai S., Tamura A., Saito R., Kawahara M., Maruyama Y. (2010). Gender, histology, and time of diagnosis are important factors for prognosis: Analysis of 1499 never-smokers with advanced non-small cell lung cancer in Japan. J. Thorac. Oncol..

[10] Pao W., Miller V., Zakowski M., Doherty J., Politi K., Sarkaria I., Singh B., Heelan R., Rusch V., Fulton L. (2004). EGF receptor gene mutations are common in lung cancers from “never smokers” and are associated with sensitivity of tumors to gefitinib and erlotinib. Proc. Natl. Acad. Sci. USA.

[11] Samet J.M. (2018). Lung Cancer, Smoking, and Obesity: It’s Complicated. J. Natl. Cancer Inst..

[12] Samet J.M. (2013). Tobacco smoking: The leading cause of preventable disease worldwide. Thorac. Surg. Clin..

[13] Lynch T.J., Bell D.W., Sordella R., Gurubhagavatula S., Okimoto R.A., Brannigan B.W., Harris P.L., Haserlat S.M., Supko J.G., Haluska F.G. (2004). Activating mutations in the epidermal growth factor receptor underlying responsiveness of non-small-cell lung cancer to gefitinib. N. Engl. J. Med..

[14] Koo L.C., Ho J.H. (1990). Worldwide epidemiological patterns of lung cancer in nonsmokers. Int. J. Epidemiol..

[15] Nordquist L.T., Simon G.R., Cantor A., Alberts W.M., Bepler G. (2004). Improved survival in never-smokers vs current smokers with primary adenocarcinoma of the lung. Chest.

[16] Paez J.G., Janne P.A., Lee J.C., Tracy S., Greulich H., Gabriel S., Herman P., Kaye F.J., Lindeman N., Boggon T.J. (2004). EGFR mutations in lung cancer: Correlation with clinical response to gefitinib therapy. Science.

[17] Malhotra J., Malvezzi M., Negri E., La Vecchia C., Boffetta P. (2016). Risk factors for lung cancer worldwide. Eur. Respir. J..

[18] Lee D.H., Lee J.S., Wang J., Hsia T.C., Wang X., Kim J., Orlando M. (2015). Pemetrexed-Erlotinib, Pemetrexed Alone, or Erlotinib Alone as Second-Line Treatment for East Asian and Non-East Asian Never-Smokers with Locally Advanced or Metastatic Nonsquamous Non-small Cell Lung Cancer: Exploratory Subgroup Analysis of a Phase II Trial. Cancer Res. Treat..

[19] Miller V.A., Kris M.G., Shah N., Patel J., Azzoli C., Gomez J., Krug L.M., Pao W., Rizvi N., Pizzo B. (2004). Bronchioloalveolar pathologic subtype and smoking history predict sensitivity to gefitinib in advanced non-small-cell lung cancer. J. Clin. Oncol..

[20] Shigematsu H., Lin L., Takahashi T., Nomura M., Suzuki M., Wistuba I.I., Fong K.M., Lee H., Toyooka S., Shimizu N. (2005). Clinical and biological features associated with epidermal growth factor receptor gene mutations in lung cancers. J. Natl. Cancer Inst..

[21] Islami F., Torre L.A., Jemal A. (2015). Global trends of lung cancer mortality and smoking prevalence. Transl. Lung Cancer Res..

[22] Choi J.R., Park S.Y., Noh O.K., Koh Y.W., Kang D.R. (2016). Gene mutation discovery research of non-smoking lung cancer patients due to indoor radon exposure. Ann. Occup. Environ. Med..

[23] Secretan B., Straif K., Baan R., Grosse Y., El Ghissassi F., Bouvard V., Benbrahim-Tallaa L., Guha N., Freeman C., Galichet L. (2009). A review of human carcinogens--Part E: Tobacco, areca nut, alcohol, coal smoke, and salted fish. Lancet Oncol..

[24] Cheng Y.W., Chiou H.L., Sheu G.T., Hsieh L.L., Chen J.T., Chen C.Y., Su J.M., Lee H. (2001). The association of human papillomavirus 16/18 infection with lung cancer among nonsmoking Taiwanese women. Cancer Res..

[25] Liang H.Y., Li X.L., Yu X.S., Guan P., Yin Z.H., He Q.C., Zhou B.S. (2009). Facts and fiction of the relationship between preexisting tuberculosis and lung cancer risk: A systematic review. Int. J. Cancer.

[26] Li X., Li J., Wu P., Zhou L., Lu B., Ying K., Chen E., Lu Y., Liu P. (2018). Smoker and non-smoker lung adenocarcinoma is characterized by distinct tumor immune microenvironments. Oncoimmunology.

[27] Brunelli M., Bria E., Nottegar A., Cingarlini S., Simionato F., Calio A., Eccher A., Parolini C., Iannucci A., Gilioli E. (2012). True 3q chromosomal amplification in squamous cell lung carcinoma by FISH and aCGH molecular analysis: Impact on targeted drugs. PLoS ONE.

[28] Cancer Genome Atlas Research Network (2012). Comprehensive genomic characterization of squamous cell lung cancers. Nature.

[29] Forbes S.A., Beare D., Gunasekaran P., Leung K., Bindal N., Boutselakis H., Ding M., Bamford S., Cole C., Ward S. (2015). COSMIC: Exploring the world’s knowledge of somatic mutations in human cancer. Nucleic Acids Res..

[30] Baldassarri M., Fallerini C., Cetta F., Ghisalberti M., Bellan C., Furini S., Spiga O., Crispino S., Gotti G., Ariani F. (2018). Omic Approach in Non-smoker Female with Lung Squamous Cell Carcinoma Pinpoints to Germline Susceptibility and Personalized Medicine. Cancer Res. Treat..

[31] Vanni I., Coco S., Bonfiglio S., Cittaro D., Genova C., Biello F., Mora M., Rossella V., Dal Bello M.G., Truini A. (2016). Whole exome sequencing of independent lung adenocarcinoma, lung squamous cell carcinoma, and malignant peritoneal mesothelioma: A case report. Medicine (Baltimore).

[32] Tong L. (2005). Acetyl-coenzyme A carboxylase: Crucial metabolic enzyme and attractive target for drug discovery. Cell Mol. Life Sci..

[33] Garassino M.C., Crino L., Catino A., Ardizzoni A., Cortesi E., Cappuzzo F., Bordi P., Calabrò L., Barbieri F., Santo A. (2018). Nivolumab in never-smokers with advanced squamous non-small cell lung cancer: Results from the Italian cohort of an expanded access program. Tumour Biol..

[34] Bonanno L., Calabrese F., Nardo G., Calistri D., Tebaldi M., Tedaldi G., Polo V., Vuljan S., Favaretto A., Conte P. (2016). Morphological and genetic heterogeneity in multifocal lung adenocarcinoma: The case of a never-smoker woman. Lung Cancer.

[35] Okazaki I., Ishikawa S., Sohara Y. (2014). Genes associated with succeptibility to lung adenocarcinoma among never smokers suggest the mechanism of disease. Anticancer Res..

[36] Yang P., Cerhan J.R., Vierkant R.A., Olson J.E., Vachon C.M., Limburg P.J., Parker A.S., Anderson K.E., Sellers T.A. (2002). Adenocarcinoma of the lung is strongly associated with cigarette smoking: Further evidence from a prospective study of women. Am. J. Epidemiol..

[37] Zhang Y., Kent J.W., Olivier M., Ali O., Cerjak D., Broeckel U., Abdou R.M., Dyer T.D., Comuzzie A., Curran J.E. (2013). A comprehensive analysis of adiponectin QTLs using SNP association, SNP cis-effects on peripheral blood gene expression and gene expression correlation identified novel metabolic syndrome, MetS) genes with potential role in carcinogenesis and systemic inflammation. BMC Med. Genomics.

[38] Mazieres J., Rouquette I., Lepage B., Milia J., Brouchet L., Guibert N., Beau-Faller M., Validire P., Hofman P., Fouret P. (2013). Specificities of lung adenocarcinoma in women who have never smoked. J. Thorac. Oncol..

[39] Russo A., Autelitano M., Bisanti L. (2008). Metabolic syndrome and cancer risk. Eur. J. Cancer.

[40] Flegal K.M., Graubard B.I., Williamson D.F., Gail M.H. (2007). Cause-specific excess deaths associated with underweight, overweight, and obesity. JAMA.

[41] Lee J.Y., Jeon I., Lee J.M., Yoon J.M., Park S.M. (2013). Diabetes mellitus as an independent risk factor for lung cancer: A meta-analysis of observational studies. Eur. J. Cancer.

[42] Cannioto R., Etter J.L., LaMonte M.J., Ray A.D., Joseph J.M., Al Qassim E., Eng K.H., Moysich K.B. (2018). Lifetime Physical Inactivity is Associated with Lung Cancer Risk and Mortality. Cancer Treat. Res. Commun..

[43] Patel A.V., Carter B.D., Stevens V.L., Gaudet M.M., Campbell P.T., Gapstur S.M. (2017). The relationship between physical activity, obesity, and lung cancer risk by smoking status in a large prospective cohort of US adults. Cancer Causes Control..

[44] Calle E.E., Rodriguez C., Jacobs E.J., Almon M.L., Chao A., McCullough M.L., McCullough M.L., Feigelson H.S., Thun M.J. (2002). The American Cancer Society Cancer Prevention Study II Nutrition Cohort: Rationale, study design, and baseline characteristics. Cancer.

[45] Hassfjell C.S., Grimsrud T.K., Standring W.J.F., Tretli S. (2017). Lung cancer incidence associated with radon exposure in Norwegian homes. Tidsskr. Nor. Laegeforen..

[46] Kettunen E., Hernandez-Vargas H., Cros M.P., Durand G., Le Calvez-Kelm F., Stuopelyte K., Jarmalaite S., Salmenkivi K., Anttila S., Wolff H. (2017). Asbestos-associated genome-wide DNA methylation changes in lung cancer. Int. J. Cancer.

[47] Santarelli L., Gaetani S., Monaco F., Bracci M., Valentino M., Amati M., Rubini C., Sabbatini A., Pasquini E., Zanotta N. (2019). Four-miRNA Signature to Identify Asbestos-Related Lung Malignancies. Cancer Epidemiol. Biomarkers Prev..

[48] Goncalves R.B., Coletta R.D., Silverio K.G., Benevides L., Casati M.Z., da Silva J.S., Nociti F.H. (2011). Impact of smoking on inflammation: Overview of molecular mechanisms. Inflamm. Res..

[49] Quail D.F., Joyce J.A. (2013). Microenvironmental regulation of tumor progression and metastasis. Nat. Med..

[50] Fridman W.H., Pages F., Sautes-Fridman C., Galon J. (2012). The immune contexture in human tumours: Impact on clinical outcome. Nat. Rev. Cancer.

[51] Kawai O., Ishii G., Kubota K., Murata Y., Naito Y., Mizuno T., Aokage K., Saijo N., Nishiwaki Y., Gemma A. (2008). Predominant infiltration of macrophages and CD8+) T Cells in cancer nests is a significant predictor of survival in stage IV nonsmall cell lung cancer. Cancer.

[52] Trojan A., Urosevic M., Dummer R., Giger R., Weder W., Stahel R.A. (2004). Immune activation status of CD8+ T cells infiltrating non-small cell lung cancer. Lung Cancer.

[53] Hiraoka K., Miyamoto M., Cho Y., Suzuoki M., Oshikiri T., Nakakubo Y., Itoh T., Ohbuchi T., Kondo S., Katoh H. (2006). Concurrent infiltration by CD8+ T cells and CD4+ T cells is a favourable prognostic factor in non-small-cell lung carcinoma. Br. J. Cancer.

[54] Tobin N.P., Foukakis T., De Petris L., Bergh J. (2015). The importance of molecular markers for diagnosis and selection of targeted treatments in patients with cancer. J. Intern. Med..

[55] Popper H.H., Ryska A., Timar J., Olszewski W. (2014). Molecular testing in lung cancer in the era of precision medicine. Transl. Lung Cancer Res..

[56] Sampsonas F., Ryan D., McPhillips D., Breen D.P. (2014). Molecular testing and personalized treatment of lung cancer. Curr. Mol. Pharmacol..

[57] Williams A.S., Greer W., Bethune D., Craddock K.J., Flowerdew G., Xu Z. (2016). ALK+ lung adenocarcinoma in never smokers and long-term ex-smokers: Prevalence and detection by immunohistochemistry and fluorescence in situ hybridization. Virchows Arch..

[58] Rodig S.J., Mino-Kenudson M., Dacic S., Yeap B.Y., Shaw A., Barletta J.A., Stubbs H., Law K., Lindeman N., Mark E. (2009). Unique clinicopathologic features characterize ALK-rearranged lung adenocarcinoma in the western population. Clin. Cancer Res..

